# Extracting the Dynamic Magnetic Contrast in Time-Resolved X-Ray Transmission Microscopy

**DOI:** 10.3390/nano9070940

**Published:** 2019-06-28

**Authors:** Taddäus Schaffers, Thomas Feggeler, Santa Pile, Ralf Meckenstock, Martin Buchner, Detlef Spoddig, Verena Ney, Michael Farle, Heiko Wende, Sebastian Wintz, Markus Weigand, Hendrik Ohldag, Katharina Ollefs, Andreas Ney

**Affiliations:** 1 Institute of Semiconductor and Solid State Physics, Johannes Kepler University Linz, 4040 Linz, Austria; 2 Faculty of Physics and Center for Nanointegration Duisburg-Essen (CENIDE), University of Duisburg-Essen, 47057 Duisburg, Germany; 3 Paul Scherrer Institut, 5232 Villigen PSI, Switzerland; 4 Helmholtz-Zentrum Dresden-Rossendorf, 01328 Dresden, Germany; 5 Max-Planck-Institut für Intelligente Systeme, 70569 Stuttgart, Germany; 6 Stanford Synchrotron Radiation Laboratory, SLAC National Accelerator Laboratory, Menlo Park, CA 94025, USA

**Keywords:** ferromagnetic resonance, X-ray magnetic circular dichroism, scanning transmission X-ray microscopy

## Abstract

Using a time-resolved detection scheme in scanning transmission X-ray microscopy (STXM), we measured element resolved ferromagnetic resonance (FMR) at microwave frequencies up to 10 GHz and a spatial resolution down to 20 nm at two different synchrotrons. We present different methods to separate the contribution of the background from the dynamic magnetic contrast based on the X-ray magnetic circular dichroism (XMCD) effect. The relative phase between the GHz microwave excitation and the X-ray pulses generated by the synchrotron, as well as the opening angle of the precession at FMR can be quantified. A detailed analysis for homogeneous and inhomogeneous magnetic excitations demonstrates that the dynamic contrast indeed behaves as the usual XMCD effect. The dynamic magnetic contrast in time-resolved STXM has the potential be a powerful tool to study the linear and nonlinear, magnetic excitations in magnetic micro- and nano-structures with unique spatial-temporal resolution in combination with element selectivity.

## 1. Introduction

In spintronics and magnonics, it is important to understand the magnetization dynamics on the micro- and nano-scale e.g., to be able to control the propagation of spin waves. A well-established technique to measure the dynamic magnetic behavior of a system is ferromagnetic resonance (FMR). However, classical resonator based FMR measurements are not able to detect single micro- or nano-sized objects due to their detection limit of around $10^{11}$ spins [1]. This sensitivity limit has been overcome in recent years by the development of lithographically fabricated micro-resonators [2], which are capable of measuring down to $10^{6}$ spins [3], corresponding to a single Fe-nanocube with dimensions of 30×30×30 nm${}^{3}$. Due to the lack of spatial resolution below the diameter of the micro-resonator of typically a few tens of microns, it is impossible to separate the FMR signal of a single nano-particle from the resonance signal of the whole ensemble during the homogeneous excitation of the micro-resonator cavity.

To facilitate spatial resolution, other measurement techniques have been combined with FMR excitation in order to measure a single nano-sized object in an ensemble. These measurement techniques include but are not limited to: magneto optic Kerr effect (MOKE) [4], Brillouin light scattering (BLS) [5], magnetic force microscopy (MFM) [6], scanning thermal microscopy (SThM) [7], scanning electron microscopy with polarization analysis (SEMPA) [8], and X-ray photoemission electron microscopy (X-PEEM) [9]. For most of these measurement techniques, it is not possible to measure with element selectivity (MOKE, BLS, MFM, SThM and SEMPA), while other measurement techniques like X-PEEM can only probe the surface of the sample with element selectivity. In recent years, the X-ray magnetic circular dichroism (XMCD) [10,11,12] effect has been combined with FMR in order to probe the dynamic magnetic excitation, the so-called X-ray detected ferromagnetic resonance (XFMR) [13,14], utilizing the element selectivity of the X-rays. A spatial resolution of down to 20 nm can be achieved by using a scanning transmission X-ray microscope (STXM).

By combining the micro-resonator FMR with STXM (STXM-FMR) within a synchronization scheme for the exciting microwaves and the probing X-ray photons of the synchrotron, it is possible to detect FMR with a high temporal (ps-regime) as well as spatial resolution (nm regime) [7,14,15,16]. Combining these features, STXM-FMR measurements bare the potential to significantly deepen our understanding of the dynamic magnetization of ferromagnetic heterosystems containing different chemical elements [17] as well as non-ferromagnets with induced magnetization [18].

In order to be able to draw valid conclusions from the dynamic magnetic contrast in STXM-FMR, it is necessary to perform a range of control-experiments in the first place as well as testing the robustness of the evaluation of the raw data to establish that STXM-FMR indeed provides significant information about the dynamic magnetic behavior of a given magnetic specimen based on the XMCD effect. In this paper, a range of control experiments will be presented as well as a detailed analysis of the separation of the true magnetic contrast from background effects. The obtained results allow for reliably image homogeneous and inhomogeneous magnetic excitations in magnetic micro-stuctures with very high spatio-temporal resolution. Furthermore, it is possible to obtain quantitative information about the local precession angle in FMR and its relative phase within a given STXM-FMR experiment.

## 2. Experimental Details

**Figure 1:**
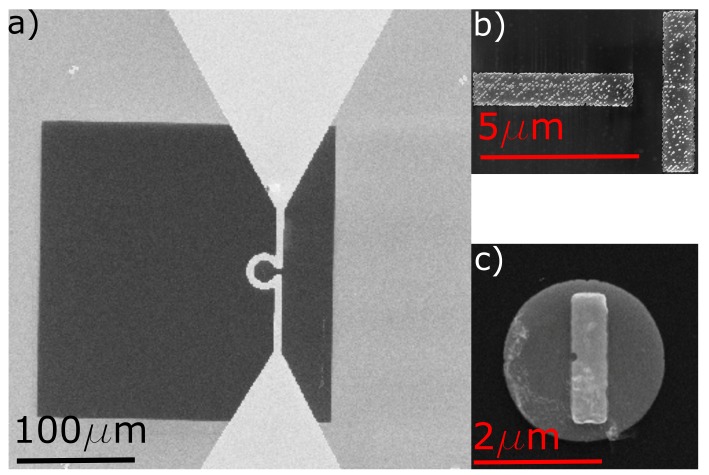
(**a**) Scanning electron microscope (SEM) image of the strip-line resonator on top of a SiN-membrane. For this work, two different sample systems were chosen. In (**b**), two perpendicular Py stripes (“T”-sample) are shown, while in (**c**)) the Py-Co disk stripe sample can be seen.

The magnetic specimen is placed inside a micro-resonator and microwaves are used to excite the FMR. The micro-resonator is fabricated on a 200 nm thick, 250 × 250 μm${}^{2}$ large silicon nitride membrane suspended by a 5 × 10 mm${}^{2}$ silicon frame of high resistivity. In a first step, the magnetic specimen is fabricated on the SiN-membrane using electron beam lithography (EBL). Two different designs for the magnetic specimen were made. The first one consists of two perpendicular permalloy (Py) stripes with dimensions of 5 × 1 × 0.03 μm${}^{3}$ (see Figure 1b), which are deposited using magnetron sputtering at room temperature and capped with aluminum. The second sample system is a combination of a Py disk with a Co stripe. For this in a first step, a Py disk with a diameter of 2.6 μm and a thickness of 30 nm is fabricated. With a second EBL step, a Co stripe with lateral dimensions of 2 × 0.6 μm${}^{2}$ and a thickness of 30 nm is placed on top of the Py disk (see Figure 1c). Finally, the micro-resonator is patterned around the magnetic specimen using optical lithography (OL), leaving the sample inside the Ω shaped resonator loop in Figure 1a. The gold used to produce the micro-resonator has a thickness of 600 nm and an additional 5 nm of titanium is used as an adhesion layer. Both materials are deposited by thermal evaporation.

By utilizing the STXM, it is possible to measure with a spatial resolution of 35 nm at SSRL and 20 nm at the MAXYMUS beamline at BESSY II, achieved by focusing the X-rays onto the magnetic specimen using a zone-plate. To measure the STXM-FMR at the Stanford Synchrotron Radiation Lightsource (SSRL), beamline 13.1, the FMR excitation needs to be synchronized to the bunch frequency of the synchrotron, thus enabling us to measure the FMR precession with time resolution. The stroboscopic time resolution for the STXM-FMR measurement is achieved by phase locking the GHz microwave frequency to the 476.315 MHz bunch frequency of the SSRL synchrotron. Furthermore, a PIN-diode was installed to switch the microwave on and off with the synchrotron revolving frequency of 1.28 MHz. This comparison of X-ray transmission detected with and without applied microwave power allows for detecting very small changes in the X-ray transmission as a result of the magnetization precession. A fast avalanche photo diode (APD) detects the transmitted X-ray photons behind the sample. The APD signal is finally stored in 12 different channels. Each of these channels corresponds to the signal of a specific group of X-ray pulses. The first six channels are used for the APD signal of the transmitted X-rays with applied microwave, while the second six channels are used to measure the X-ray transmission without applied microwave. For the first six channels, the magnetization inside the sample is precessing, while the magnetization is static for the second six channels. Each of these channels corresponds to a specific relative phase of the FMR precession with respect to the microwave excitation. The latter non-precessing channels are crucial for eliminating the influence of the filling pattern of the bunch train of electron buckets on the resulting STXM images. The six different channels correspond to six specific bunches that are phase shifted each by 60${}^{{}^{\circ}}$, with respect to the microwave frequency of up to 9.6 GHz. Therefore, the six phases correspond to time resolved snap-shot images which are separated by 17.4 ps, and comprise one full precession cycle of the magnetization. One should note that each X-ray flash has a pulse duration of 50 ps, which fundamentally limits the attainable temporal resolution. Additional information regarding the synchronization scheme and the X-ray microscope can be found in [7,15].

A similar approach for measuring the dynamic magnetization in an FMR experiment with spatial and temporal resolution has been implemented at the Maxymus endstation at BESSY II [19,20]. There are two moderate differences with respect to the SSRL experiment: one is that the BESSY II operation frequency is appx. 500 MHz, corresponding to a repetition period of the probing X-ray flashes of 2 ns. Secondly, the signal is recorded only for the microwave on (precessing) case. Therefore, on the one hand, it is not necessary to excite the sample at direct higher harmonic frequencies of the synchrotron and thus the exciting frequencies can be chosen more freely to f = 500 MHz·M/N, depending on the number of detection channels used (N) and a selectable integer multiplier M [16]. Here, N for most cases is also equal to the number of simultaneously acquired excitation phases (not limited to 6). Since the non-precessing magnetization (microwave off) cannot be used as a baseline for comparison only the transmitted intensity ratio of each channel with respect to the temporal average I(t)/< I >t can be used for extracting the dynamic magnetic contrast associated with the precession of magnetization. In addition, for each channel on average, all bunches contribute equally so that the filling pattern of the ring is averaged out.

## 3. Contrast Mechanism

For a better understanding of the measured STXM-FMR data, we briefly discuss the underlying physical effect, which yields the dynamic magnetic contrast images.

### 3.1. X-ray Absorption

**Figure 2:**
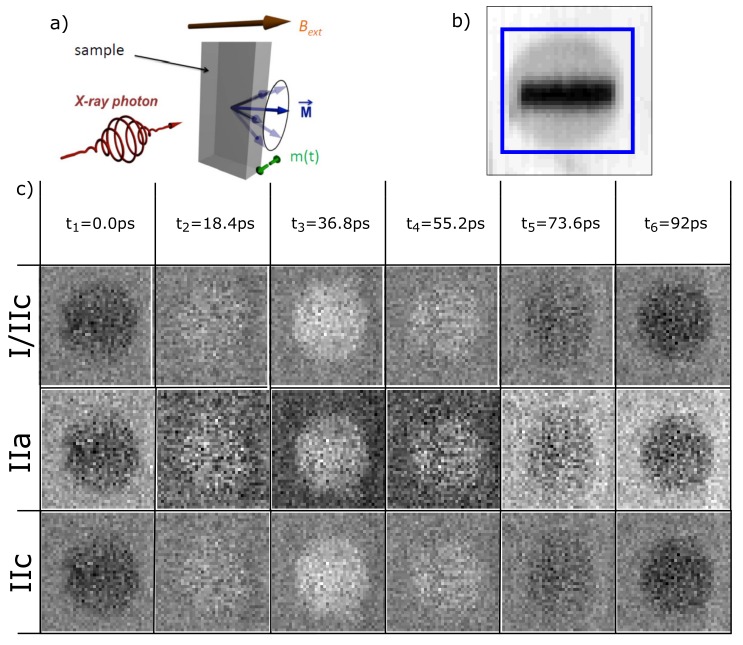
(**a**) sketch of the experimental geometry. The polarized photons hit the sample at normal incidence; the static magnetization is oriented parallel to the external magnetic field $B_{ext}$. At ferromagnetic resonance, *M* precesses and a time-dependent out-of-plane component m(t) exists; (**b**) the chemical contrast image of the disk stripe sample measured at the Ni-$L_{3}$-edge. Red and blue frames define different regions of interest; (**c**) representation of different evaluation methods for the six phases of the magnetization precession: in the absorption coefficient, the difference is shown obtaining the background corrected microwave on and off measurement. IIa is the ratio between the not background corrected microwave on and off measurement. Applying a background correction to IIa, the images labelled IIb are generated—for details, see text.

**Figure 3:**
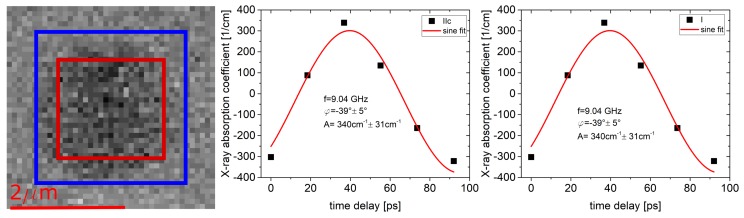
By averaging over the sample in the six different phases for evaluation method IIc (left side) and I (right side) from Figure 2, one can obtain the curves shown. Both were fitted with a sine function due to the sinusoidal behavior of the exciting microwave. The frequency of this sinusoidal was given by the microwave frequency applied to the system which in this case was 9.04 GHz.

The transmission of electromagnetic radiation through any material is described by Beer–Lambert‘s law [21]. In an STXM, the transmitted X-ray intensity *I* is detected in normal incidence. This transmitted intensity is controlled by the composite X-ray absorption (XA) coefficient of the entire sample (magnetic specimen and SiN-membrane). Tuning the photon energy to any characteristic core-level excitation results in the well-known element selectivity of XA measurements. However, the above-mentioned law only considers a single layer system. In an STXM-FMR experiment, the sample consists at least of a two layers with distinct properties, since any magnetic specimen is supported by the SiN-membrane through which the X-rays need to be transmitted as well. In order to include this second layer, a Beer-Lambert‘s law needs to be modified [21]:

(1) $$I_{s/m}=I_{0}e^{-(\mu_{s}t_{s}+\mu_{m}t_{m})},$$

where $t_{s},t_{m}$ are the thicknesses and $\mu_{s},\mu_{m}$ are the absorption coefficients of the magnetic specimen and SiN-membrane, respectively, and $I_{0}$ is the incoming intensity. In any sample, one can find areas where the X-rays only transmit through the SiN-membrane while in other regions the X-rays are transmitted through the SiN-membrane plus the magnetic specimen, which can also consist of more than one layer that would be added to the exponent in Equation (1). Therefore, the dynamic magnetic contrast can be separated from the background transmission through the SiN-membrane by defining the respective regions of interests (RoI) from the individual, time-averaged z-contrast STXM-FMR images like in Figure 2b or Figure 3.

### 3.2. XMCD Effect in STXM-FMR

It is well-known that for circular polarized X-rays at the $L_{3/2}$-edges of ferromagnetic transition metals the XA coefficient μ depends on the relative orientation of the magnetization *M* and the polarization vector $\sigma^{+}$ and $\sigma^{-}$ of the synchrotron light, respectively, called the XMCD effect [11,12]:

(2) $$I\left(\sigma^{+/-}\right)=I_{0}e^{{-\mu }^{+/-}\cdot t},$$

where $\mu^{+/-}$ is the absorption coefficient with the magnetization *M* parallel/antiparallel to the polarization vector $\sigma^{+/-}$. The XMCD in XA spectroscopy is commonly defined as the difference in absorption spectra between parallel and antiparallel orientation, i.e., for XMCD in transmission geometry $(\mu^{+}-\mu^{-})\cdot t=\Delta \mu \cdot t$. In the following, the usual spectroscopic definition of the XMCD effect will be used rather than the so-called XMCD asymmetry $\left(I\left(\sigma^{+}\right)-I\left(\sigma^{-}\right)\right)/\left(I\left(\sigma^{+}\right)+I\left(\sigma^{-}\right)\right)$ which can also be found throughout the literature. However, other than in normal XA spectroscopy in STXM-FMR, the images are only taken for a fixed photon energy for which the XMCD signal is maximal.

For a single STXM-FMR image, neither the circular polarization nor the direction of the magnetization is reversed. This is due to the fact that the static magnetizatioj *M* is parallel to the external static magnetic field $B_{ext}$ which in turn is perpendicular to the incident light, i.e., a transverse geometry with X-ray beam normal to the surface and *M* in the sample plane is chosen—see sketch in Figure 2a. Therefore, only a small, dynamic out of plane component m(t) of the precessing magnetization is accessible by the XMCD effect. At the SSRL, the difference between microwave on and off is therefore the difference between the precessing (= finite m(t) out-of-plane) and non-precessing case (m(t)=0 for all *t*) is recorded via the XMCD effect. In contrast, in the detection scheme at BESSY II, the average over all images corresponding to one full cycle of precession is taken, in which the dynamic magnetization component averages out and is subsequently subtracted from each individual phase. Both methods are equivalent in the sense that the XMCD effect only senses the finite out-of-plane component of the precessing magnetization m(t) which requires the time-resolved detention scheme which records snapshot images of m(t) at different points in time throughout a full precession cycle—for details, see [15].

## 4. Analysis of STXM-FMR Measurements

In light of the preceding discussion, evaluation methods for the extraction of quantitative information from the STXM-FMR data will be presented, with special attention to how to extract the dynamic contribution m(t) of the magnetic specimen. Additionally, it is possible to quantify the opening angle of the magnetization precession in FMR directly from the change in absorption coefficient during a full precession cycle.

### 4.1. Raw Data Treatment

To eliminate the second absorption coefficient $\mu_{m}$ in Equation (1), the raw data needs to be corrected by the SiN-membrane background. Thus, the absorption coefficient of the magnetic specimen alone can be investigated. For that, we average the transmission signal over the area of only the SiN-membrane for each of the 12 images (six phases with microwave on $I_{m}^{on}$ and six phases with microwave off $I_{m}^{off}$) separately. This corresponds to the area outside the blue box in Figure 2b. Each individual image is then divided by its respective averaged transmission value of the SiN-membrane. The resulting transmission $I^{on}$ and $I^{off}$ then contains exclusively the information about the absorption coefficient $\mu_{s}$ of the magnetic specimen:

(3) $$I_{s}^{on}=\frac{I_{s/m}^{on}}{I_{m}^{on}}=e^{\begin{array}{c} - \end{array}\mu_{s}^{on}\cdot t_{s}}\;I_{s}^{off}=\frac{I_{s/m}^{off}}{I_{m}^{off}}=e^{\begin{array}{c} - \end{array}\mu_{s}^{off}\cdot t_{s}}.$$

This procedure also eliminates random fluctuations of the incoming intensity $I_{0}$ for each phase. Subsequently, the dynamic magnetic contrast is derived by taking the natural logarithm of the ratio of the precessing (microwave on) versus non-precessing (microwave off) case to obtain Δμ corresponding to the difference in absorption coefficient equivalent to the usual definition of the XMCD effect:

(4) $$ln\left(\frac{I_{s}^{on}}{I_{s}^{off}}\right)=\left(\mu_{s}^{off}-\mu_{s}^{on}\right)\cdot t=\Delta \mu \cdot t,$$

where *t* is the thickness of the magnetic specimen. The resulting dynamic magnetic contrast Δμ·t of the Py disk recorded at the Ni $L_{3}$-edge at 9.04 GHz is shown for all six phases in Figure 2c, row I/IIc. It is clearly visible that only the contrast of the Py disk reverses during a full measurement cycle representing the perpendicular component of the high-frequency magnetization m(t), while the background stays constant.

However, one can change the sequence of extracting Δμ·t and take a closer look at each individual step. First, the ratio of microwave on and microwave off is taken and all six phases are displayed in Figure 2 row IIa. It is obvious that the background corresponding to the SiN-membrane oscillates as well, which will be discussed further below. In a second step, the influence of the oscillating background is corrected as mentioned before by dividing each phase with the respective averaged transmission of the SiN-membrane derived outside the blue area. The resulting six phases are shown in Figure 2c, row IIb and already compare well with the full analysis of I, revealing no visible background oscillation.

However, the images of IIb do not directly reflect the numerical values of Δμ·t. Taking the natural logarithm of IIb, one obtains the numerically identical six phase images as shown in Figure 2c, row I/IIc. A direct comparison between IIb and I/IIc reveals that the qualitative behavior of the dynamic magnetic contrast is identical. However, only IIc shall be mathematically equivalent to the full analysis in I. Both evaluations depend on the selection of the RoI from which the background of the SiN-membrane is derived, see Figure 3 below.

To verify if the sequence of the evaluation steps indeed yield the same results, the quantitative outcome of methods I and IIc are compared in Figure 3. In the STXM-FMR image, the area outside the blue box defines the RoI used for determining the background of the SiN-membrane. The red box indicates the RoI which is used for determining the average Δμ·t of the magnetic specimen. To derive the absorption coefficient Δμ at Ni $L_{3}$-edge, the resulting averaged value has to be divided by the effective thickness t=24 nm, since the Py film is 30 nm thick and contains 80% nickel; note that the non-resonant XA of the iron can be excluded due to the ratio between the measurements with and without applied microwave power. The two panels show the averaged values (symbols) of the six phases for method I (right) and IIc (left) reflecting the dynamic magnetic contrast of the homogeneous excitation, i.e., uniform mode of the Py disk. The sine fits (solid lines) are done for the fixed frequency of the exciting microwave of 9.04 GHz while amplitude *A* and phase φ are fitting parameters. Indeed, both methods reveal identical numerical values for A=Δμ and φ of (340±31) cm ${}^{-1}$ and $-39^{{}^{\circ}}\pm 5^{{}^{\circ}}$, respectively. The quantitative numerical values will be discussed in the following.

### 4.2. Precession Angle

The first quantity that can be extracted from a STXM-FMR experiment is the amplitude *A* corresponding to the dynamic magnetic contrast Δμ. For a known thickness *t* of the magnetic specimen, one can extract the opening angle θ of the precessing magnetization. Other than for the phase φ, the amplitude *A* and thus Δμ can be compared between different samples. For that, a usual XMCD experiment is carried out on a specimen of known thickness *d* where $\Delta \mu_{XMCD}$ is derived as the difference in absorption with the magnetization fully parallel and antiparallel to the *k* vector of the X-rays, yielding $\Delta \mu_{abs}=\Delta \mu_{XMCD}/d$. One should keep in mind that in an XMCD experiment the magnetization is fully reversed while, in the STXM-FMR measurement, microwave off corresponds to the fully perpendicular case. Therefore, 2A has to be taken when comparing with $\Delta \mu_{abs}$. Geometrical considerations yield the full opening angle of the precession cone corresponding to 2θ, therefore yielding:

(5) $$sin\left(2\theta \right)=\frac{2A}{\Delta \mu_{abs}}.$$

Here, 2A is (680±31) cm${}^{-1}$ and $\Delta \mu_{abs}\approx$ 200,000$\;{\mathrm{cm}}^{-1}$, which yields an opening angle of $\theta \;=\;0.10^{{}^{\circ}}\pm 0.01^{{}^{\circ}}$. As already pointed out before [15], one has to consider the effect of the pulse length of the X-rays on the measured intensity. Comparing the pulse length of 50 ps with the duration of a full precession cycle of 110.4 ps makes clear that each light pulse averages over a substantial fraction of the sine-like contrast variation in time which yields a reduction factor of 1.5 compared to an ideal sampling of the dynamic magnetic contrast. Therefore, the actual opening angle for this FMR measurement is $\theta =0.15^{{}^{\circ}}\pm 0.02^{{}^{\circ}}$, which is of the same order as the previously reported opening angle of $0.1^{{}^{\circ}}$ for a Co-stripe [15]. It has to be taken into consideration that the obtained opening angle of the FMR is only the out-of-plane angle, which in turn can differ from the in plane angle due to the magnetic anisotropy of the thin film sample. In addition, the measured opening angle naturally depends also on the microwave power applied to the magnetic specimen. This cannot be measured directly and it differs from sample to sample since the contact from the standard SMA cabling to the lithographically fabricated microresonator are so far not perfectly impedance matched and thus the entire system can have different transmissivity/reflectivity for microwaves leading to a variation of the microwave power at the sample for different specimen.

### 4.3. Origin of the Background Signal

The origin of the background oscillation visible in Figure 2 IIa needs to be discussed. One has to keep in mind that the output signal of the avalanche photo diode is amplified by a factor of 1000 (60 dB) to be detected. Therefore, it is very sensitive to issues with the pre-amplification. The cables inside the STXM (power supply for the APD and signal output of the APD) can act as antennas for standing waves generated by the microwave excitation of the sample. This can cause false positives/negatives depending of the phase of the microwave with regard to the photon arrival time, which can be misinterpreted as bulk (low spatial frequency) dynamics. This is an issue since microvolts of induced voltage by the microwave can be amplified to a “photon” level in the signal output of the APD. Furthermore, common detection methods can only detect one photon per bunch. Multi photon events only register as single events. This creates a nonlinear, detector response that gets more pronounced for higher count rates, and can interfere with normalization of dynamic contrast when imaging samples with big static contrast. While the signal in dark areas (magnetic specimen) is linear, the signal in bright areas (SiN-membrane) is compressed, thus the normalization algorithms that work by averaging obtain a skewed response that can create false dynamic contrast proportional to the static contrast. However, if the dynamic contrast reverses when switching the helicity of the light, i.e., the fitted phase between a STXM-FMR measurement with $\sigma^{+}$ and $\sigma^{-}$ is 180${}^{{}^{\circ}}$, it can be concluded that the observed signal is a consequence of the dynamic magnetic response of the system according to the XMCD effect and thus the dynamic contrast is of magnetic nature and thus stems from the external excitation of the magnetization generated by the microwave. Before the contrast reversal is demonstrated experimentally, the physical meaning of the phase of the sine fit shall be addressed.

### 4.4. Absolute vs. Relative Phase

The absolute phase should be measured between the precessing magnetization and the arrival of the X-ray pulse. However, this is complicated due to several issues. First, as in any resonance experiment, there is a phase difference between the microwave excitation and the precessing magnetization. Second, the phase of the X-ray pulse with respect to the microwave excitation cannot be determined directly. For the synchronization between microwave and X-ray pulse, only the driving frequency of the rf-cavity is available. This frequency determines the internal time structure of the synchrotron beam, i.e., it splits the electron beam into individual bunches. Inside the undulator, each of the bunches emits an X-ray pulse of fixed duration. Therefore, the travel time of the electron bunches from the cavity to the undulator as well as the travel time of the X-ray pulse from the undulator to the sample have to be taken into account. These are in principle known and should be fixed values for a given synchrotron. In addition, the length of the used cabling has an influence on the phase as well and this changes when the microwave set-up including sample and micro-resonator is physically changed. In a practical experiment, the fitted phase φ is only a relative number and comprises all the above factors. Therefore, it can only be compared as long as the entire microwave set-up as well as the excitation frequency of the STXM-FMR experiment is not changed. This implies that it is only possible to compare relative phases within the same sample and not between different samples. In other words, the obtained phase $\varphi =-39^{{}^{\circ}}\pm$ 5${}^{{}^{\circ}}$ is basically meaningless for comparing different sample measured in the STXM-FMR. However, the relative phase of different measurements using the same parameters and sample upon, e.g., the reversal of the helicity of the light can be compared and—according to the XMCD effect—should be 180${}^{{}^{\circ}}$.

## 5. Experimental Verification of the Magnetic Nature of the Dynamic Contrast

Having discussed the small variation of the absorption coefficient during precession with a small opening angle together with the presence of a rather pronounced background signal of the SiN-membrane, it is important to investigate the behavior of the dynamic magnetic contrast upon reversal of the helicity of the light to assure that a true XMCD effect is indeed observed.

### 5.1. Contrast Reversal with Helicity

**Figure 4:**
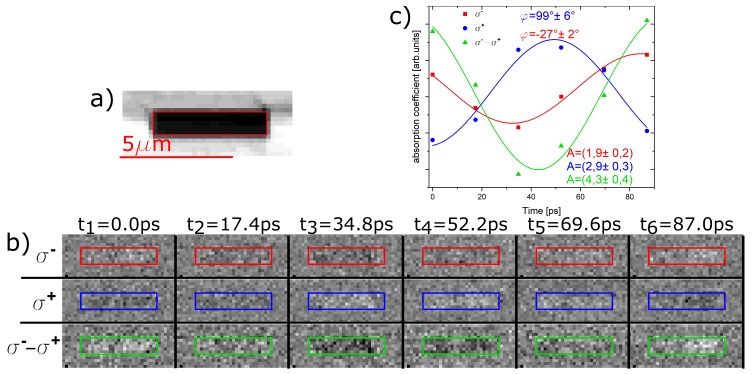
(**a**) chemical contrast picture of the Py stripe. In (**b**), two measurements of the dynamic magnetic contrast with different X-ray polarization taken at the Ni-$L_{3}$-edge are shown as well as the difference between the two measurements; (**c**) shows the average transmission intensity of the X-rays through the stripe sample for the three cases shown in (**b**). The averaged data was fitted with a microwave frequency of 9.61 GHz.

As a first test experiment, two STXM-FMR measurements at 9.61 GHz were done using $\sigma^{+}$ and $\sigma^{-}$ polarized X-rays at the Ni $L_{3}$-edge of the horizontal Py stripe of the T sample shown in Figure 1b. In Figure 4a, the chemical contrast is shown while the individual six phases with $\sigma^{+}$ (blue) and $\sigma^{-}$ (red) light are displayed in Figure 4b, top two rows. All contrast variations in (b) are shown on the same scale in order to emphasize the difference in Δμ·t for the different measurements. Figure 4c collates the averaged dynamic magnetic contrast for all six phases derived by averaging over the respective marked areas. The RoIs were identified using the chemical contrast image in Figure 4a as indicated by the red box. The same colour scheme was used for the averaged intensities of the two measurements shown in Figure 4c.

As one can clearly see in the individual phase images, there is a phase difference of $-27^{{}^{\circ}}\pm 2^{{}^{\circ}}$ for $\sigma^{-}$ and $+99^{{}^{\circ}}\pm 6^{{}^{\circ}}$ for $\sigma^{+}$ light. Note that the value for the phases in Figure 3 and Figure 4 differ because the samples and thus the microwave setup are different. The XMCD effect suggests that, by reversing the X-ray polarization, the relative phase should change by $180^{{}^{\circ}}$, i.e., an ideal reversal of the contrast in all six images. However, the relative phase difference between the two measurement is only $127^{{}^{\circ}}\pm 8^{{}^{\circ}}$, which is significantly smaller. This is most likely due to the experimental constraint that only six phases can be resolved because of the X-ray pulse length of 50 ps, while the time difference between the individual phases is only 17.4 ps. Therefore, the experimental uncertainty is larger than the errors from the fitting procedure, especially considering the small overall size of the dynamic contrast change. In turn, one can increase the magnetic contrast by taking the difference between the two experiments with $\sigma^{+}$ and $\sigma^{-}$ light according to usual XMCD experiments. The result is shown in Figure 4b,c (green) and it is obvious that the dynamic contrast is enhanced significantly. Nevertheless, as visible in Figure 4c, the amplitude is not increased by a factor of 2 as expected, which is due to the non-ideal reversal of the contrast as reflected by the behavior of the relative phases. Nevertheless, this is a first indication that a homogeneous FMR excitation behaves the same way as previously observed spin wave excitations [15].

### 5.2. Helicity versus Field Direction

**Figure 5:**
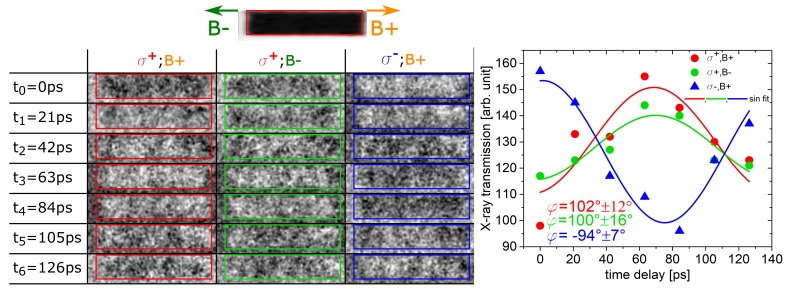
STXM-FMR measurement done at the Maxymus beamline at the Fe-$L_{3}$-edge at B+ = 60 mT and B- = −60 mT and different X-ray polarization $\sigma^{+}\;\;\sigma^{-}$. The applied microwave frequency for all measurements shown in this figure was 6.785 GHz. The left-hand side shows the chemical contrast image together with the different directions for B- and B+. The averaged area is indicated by the red box. Below, the normalized intensity (with respect to the time average state) for the seven different excitation phases (or delay times) is shown for different field directions and X-ray polarisations. The spatially averaged intensity for each of these boxes can be found on the right-hand side with their respective colour coding.

In Figure 5 a second control STXM-FMR measurement done at the Maxymus endstation at BESSY II is shown where the STXM-FMR was measured with a slightly increased phase resolution of seven images for one full precession cycle. In addition to reversing the helicity of the light, one helicity was also measured for two different external magnetic field orientations $B_{ext}$, i.e., ($\sigma^{+},B^{+}$), ($\sigma^{+},B^{-}$), and ($\sigma^{-},B^{+}$). The relative orientation of $B_{ext}$ is indicated together with the image of the chemical contrast in Figure 5, top. The RoI where the contrast is spatially averaged is indicated by the red boxes in all images to ensure that the observed averaged signal only originates from the stripe and does not contain the SiN-membrane background (see above). The time-normalized spatial average intensity for each phase of the three different measurements is shown in Figure 5. The measurement in a positive magnetic field B+ and circular polarization $\sigma^{+}$ is shown on the left side of Figure 5a. The resulting averaged normalized X-ray transmission can be found in Figure 5, where it was fitted using a sine function with the microwave frequency of 6.785 GHz. This fit yields a relative phase between the X-ray pulses and the magnetization precession of $102^{{}^{\circ}}\pm 12^{{}^{\circ}}$.

As mentioned above, the sign of the static XMCD effect reverses when the external magnetic field along an axis of sensitivity is reversed [11,12]. At first glance, this would lead to a contrast reversal for the dynamic magnetic contrast in STXM-FMR as well. However, as can be seen in Figure 5a, the contrast does not reverse when the external field is reversed (middle column). This can be explained due to the fact that the present STXM-FMR configuration senses only the transversal dynamic component of the magnetization precession and not the direction of the magnetization itself. Due to the field reversal, the magnetization still precesses around the external field with the same phase relation as before. The dynamic magnetic contrast is only dependent on the projection of the dynamic magnetization onto the X-ray k-vector. This projection in turn exhibits a cosine behaviour and thus does not depend on the sense of rotation regarding the X-ray k-vector. This is evidenced by comparing the averaged dynamic magnetic contrast for $\sigma^{+},B^{+}$ and $\sigma^{+},B^{-}$ in Figure 5. The resulting relative phase is $102^{{}^{\circ}}\pm 12^{{}^{\circ}}$ and $100^{{}^{\circ}}\pm 16^{{}^{\circ}}$, respectively, i.e., identical within error bars. In contrast, comparing $\sigma^{+}$ with $\sigma^{-}$ polarization for $B^{+}$ in Figure 5 respectively, a clear contrast reversal is seen which is reflected by the resulting relative phases of $102^{{}^{\circ}}\pm 12^{{}^{\circ}}$ and $-94^{{}^{\circ}}\pm 7^{{}^{\circ}}$. The resulting phase change is thus $196^{{}^{\circ}}\pm 19^{{}^{\circ}}$ which agrees within error bars with the expected value of $180^{{}^{\circ}}$ for the ideal XMCD effect.

### 5.3. Contrast Reversal for Spin Wave Excitations

All previous examples were obtained with a uniform magnetic excitation, i.e., the uniform mode of FMR is excited. In these experiments, the oscillating contrast of the background was attributed to direct interactions between microwaves and the APD. Nonlinear APD responses can also have a non-negligible influence on the extraction of the dynamic magnetic contrast. This is especially relevant for homogeneous excitations of the magnetic specimen. In order to exclude this, the method of choice is an inhomogeneous excitation, i.e., a spin-wave of the microstripe is excited.

**Figure 6:**
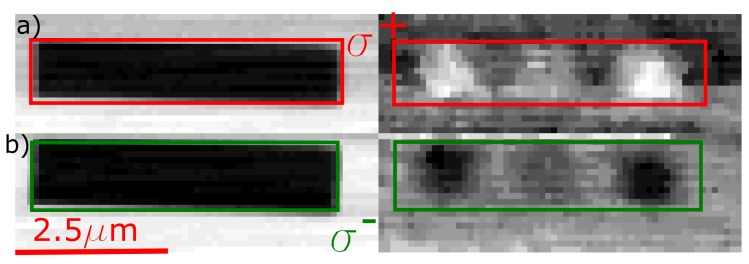
Comparison of the two circular polarizations for an inhomogeneous excitation of a T stripe. The left part shows the chemical contrast pictures for the different polarization measurements, respectively. For contrast maximization, the opposite phases of the same measurement are subtracted. The red and green boxes indicate the position of the Py stripe (extracted from the chemical contrast) for each of the two measurements to better visualize the excitation.

In Figure 6a, the STXM-FMR experiment, measured at the SSRL, of a spin wave excitation of the stripe parallel to the external magnetic field of a Py “T”-sample is shown. Details on these types of excitations go beyond the scope of this paper and will be discussed elsewhere; integral FMR measurements together with micromagnetic simulations have already identified these kind of spin wave excitations [3]. Figure 6a shows the measurement with $\sigma^{+}$ light, whereas, in b), the $\sigma^{-}$ case can be seen. On the left-hand side, the chemical contrast images are provided while on the right-hand side a single image of the dynamic magnetic contrast is displayed. In order to maximize the contrast, the difference between the same two opposite phases has been taken for both polarizations. An additional smoothing as in Ref. [7] has been carried out to better visualize the inhomogeneous excitation. One can clearly see that there are regions with a pronounced magnetic contrast to either end of the stripe while the center shows a much weaker contrast with zero contrast in between. Importantly, the regions of strong magnetic contrast clearly reverse upon reversal of the helicity of the light while in other regions the contrast remains unaffected. Therefore, the contrast reversal is also observable with respect to a non-reversing region where the overall XA does not change, underlining that the contrast mechanism is indeed of magnetic origin.

## 6. Conclusions

We have shown a way to correctly separate the quantitative pure dynamic magnetic contrast from the background signal in STXM-FMR. The opening angle of the FMR excitation of Py was determined at the Ni-L${}_{3}$-edge by evaluating the amplitude of the dynamic magnetic contrast yielding an opening angle of 0.15${}^{{}^{\circ}}$ which corresponds well with previously reported order of magnitude for Co [15]. Furthermore, by switching the polarization of the X-ray photons from $\sigma^{+}$ to $\sigma^{-}$, the dynamic magnetic contrast switches its sign for the STXM-FMR measurement. However, contrary to static classical XMCD, a reversal of the external magnetic field does not change the dynamic magnetic contrast of the STXM-FMR because of the transversal geometry. Enhancement of the signal can be achieved by measuring the STXM-FMR with different polarizations ($\sigma^{+}$ and $\sigma^{-}$). Finally, the contrast reversal upon reversal of the helicity was observable for two different STXM-FMR setups, at two different synchrotrons, as well as for an inhomogeneous excitation. This makes evident that the contrast in STXM-FMR behaves similarly under reversal of the X-ray helicity to the static XMCD effect and one can take advantage of the unique combination of element selectivity and spatio-temporal resolution in future studies of magnetically excited micro- and nano-structures.
